# Genome-Wide Analysis of the *Salmonella* Fis Regulon and Its Regulatory Mechanism on Pathogenicity Islands

**DOI:** 10.1371/journal.pone.0064688

**Published:** 2013-05-23

**Authors:** Hui Wang, Bin Liu, Quan Wang, Lei Wang

**Affiliations:** 1 TEDA School of Biological Sciences and Biotechnology, Nankai University, TEDA, Tianjin, P. R. China; 2 Tianjin Key Laboratory of Microbial Functional Genomics, Tianjin, P. R. China; 3 The Key Laboratory of Molecular Microbiology and Technology, Ministry of Education, Tianjin, P. R. China; Indian Institute of Science, India

## Abstract

Fis, one of the most important nucleoid-associated proteins, functions as a global regulator of transcription in bacteria that has been comprehensively studied in *Escherichia coli* K12. Fis also influences the virulence of *Salmonella enterica* and pathogenic *E. coli* by regulating their virulence genes, however, the relevant mechanism is unclear. In this report, using combined RNA-seq and chromatin immunoprecipitation (ChIP)-seq technologies, we first identified 1646 Fis-regulated genes and 885 Fis-binding targets in the *S. enterica* serovar Typhimurium, and found a Fis regulon different from that in *E. coli*. Fis has been reported to contribute to the invasion ability of *S. enterica*. By using cell infection assays, we found it also enhances the intracellular replication ability of *S. enterica* within macrophage cell, which is of central importance for the pathogenesis of infections. *Salmonella* pathogenicity islands (SPI)-1 and SPI-2 are crucial for the invasion and survival of *S. enterica* in host cells. Using mutation and overexpression experiments, real-time PCR analysis, and electrophoretic mobility shift assays, we demonstrated that Fis regulates 63 of the 94 *Salmonella* pathogenicity island (SPI)-1 and SPI-2 genes, by three regulatory modes: i) binds to SPI regulators in the gene body or in upstream regions; ii) binds to SPI genes directly to mediate transcriptional activation of themselves and downstream genes; iii) binds to gene encoding OmpR which affects SPI gene expression by controlling SPI regulators SsrA and HilD. Our results provide new insights into the impact of Fis on SPI genes and the pathogenicity of *S. enterica.*

## Introduction

Bacterial regulators are broadly classified into two groups-global and local, depending on the number of genes the regulator targets [1]. Nucleoid-associated proteins (NAPs) are notable among the global regulators. Most NAPs possess an ability to alter the trajectory of the DNA molecule by bending, wrapping or bridging it, which influences the transcription of numerous genes by changing the global DNA structure [2]–[5]. In addition, some NAPs also regulate specific genes by different mechanisms such as interacting with RNA polymerase and other proteins [6]. Fis, one of the best-studied NAPs, was first identified as a stimulator of inversion of the Hin invertible DNA element in *Salmonella enterica* serovar Typhimurium [7]–[10]. Fis has been studied intensively from the perspective of gene regulation and has been reported to regulate gene expression by modulating the level of DNA supercoiling in the cell and interacting with RNA polymerase at the position of its binding site [2], [11]–[14].

The effects of Fis on gene transcription have been mainly studied in *E. coli* using transcriptomics analysis and chromatin immunoprecipitation (ChIP) analysis [3], [4], [15], [16]. More than 900 genes were found to be regulated by Fis during the exponential growth stage in *E. coli* [3], [4], [17]–[19]. Genes up-regulated by Fis are involved in translation, flagellar biosynthesis and energy metabolism, while down-regulated genes are involved in stress responses, amino acid and nucleotide biosynthesis, and nutrient transport [4], [20], [21]. More than 1000 Fis-binding regions were determined, and several Fis-binding motifs were identified [3], [4], [16], [22], [23]. By comparing the genes bound by Fis with the genes regulated by Fis in *E. coli*, it was found that only a small proportion was present in both, indicating that most genes were indirectly influenced by Fis [3], [4].

A global role for Fis in transcriptional regulation in *S. enterica* serovar Typhimurium has been studied using microarrays, and Fis was found to influence 291 genes during the exponential stage [7]. Fis-binding sites on several genes such as *rpoS* and *gyrB* in *S. enterica*, through which Fis regulates these genes, have also been identified [16]. However, a genome-wide analysis of Fis-binding sites in *S. enterica* has not yet been reported. It can be speculated that the Fis-binding regions in *S. enterica* are different from those of *E. coli* for two major reasons. First, there are marked differences in the genomes of these two species. For instance, approximately 29% of the genes in *S. enterica* serovar Typhimurium LT2 (including those of pathogenicity islands, functional prophages, and plasmids, most of which are closely associated with pathogenesis), are absent from *E. coli* K12 [24], [25]. Furthermore, the genome regions present in both species share on average only 80–85% identity at the nucleotide level [24]. Second, the DNA supercoiling levels differ between the two species, and Fis binds to DNA to play an important role in the homeostasis of supercoiling [15], [26]–[28].

Besides its role in global regulation, the roles of Fis in the regulation of virulence properties in *E. coli* and *S. enterica* have been previously reported [7], [29]–[31]. For example, Fis was reported to influence the transcription of the virulence genes at the locus of enterocyte effacement (LEE) in enteropathogenic *E. coli* and therefore, to affect the invasion ability of the pathogen [29]. *S. enterica* serovar Typhimurium is a facultative intracellular pathogen that infects intestinal epithelial cells, subsequently be internalized by macrophages cells and then rapidly disseminates through the blood stream accumulating in mesenteric lymph nodes. It causes food-borne gastroenteritis in millions of people worldwide. Furthermore, its invasion process is mediated by a type III secretion system (TTSS), which is encoded by *Salmonella* pathogenicity islands (SPI)-1, and TTSS encoded by SPI-2 is responsible for delivering effector proteins to the host cell, which facilitates *S. enterica* survival and replication in host cells [25], [32]–[34]. Besides SPI-1 and SPI-2, other SPIs such as SPI-3, SPI-4 and SPI-5 also contribute to host cell invasion and intracellular pathogenesis [35]–[38]. Most of the genes in SPI-1, SPI-2, SPI-4, SPI-5, and some genes in SPI-3 have been found to be positively regulated by Fis [7], although the underlying regulatory mechanism remains to be clarified.

In this study, we determined the genome-wide distribution of Fis-binding regions in *S. enterica* LT2, analyzed the regulation of global gene transcription by Fis, and identified the molecular mechanisms by which Fis acts to influence virulence of LT2. A lower degree of concordance (23%) in the Fis-binding regions of *S. enterica* LT2 and *E. coli* K12 was found, and a new Fis-binding motif was identified in LT2 that differed from the K12 form. A large proportion (65%) of Fis-binding genes was positively regulated by Fis, which is different from the effect of Fis in *E. coli*. In addition, we found that Fis up-regulated cobalamin (B_12_) biosynthesis genes by controlling the B_12_ regulator gene, *pocR*. Using cell invasion assays, we showed that Fis enhances invasion and intracellular replication ability of LT2 within the host cell. Combining the results of ChIP-seq, RNA-seq, real-time PCR (RT-PCR), mutation, and cell infection experiments, we showed that Fis influences the expression of 63 of the 94 SPI-1 and SPI-2 genes, which are responsible for the invasion and intracellular replication of LT2. Three regulatory modes were characterized by which Fis controls SPI gene transcription: i) Fis binds to and activates SPI regulator genes (*hilC* and *ssrA*); ii) Fis binds directly to SPI genes to enhance the transcription of these genes and those downstream; iii) Fis enhances the expression of the global regulator gene *ompR* [39], which induces the expression of SPI positive regulator genes (*hilD* and *ssrA*).

## Materials and Methods

### Bacterial Strains and General Growth Conditions

Strains used in this work are listed in Table S1. Luria-Bertani broth and agar (15 g/L) were used for routine growth. Where necessary, antibiotics were used at the following final concentrations: ampicillin (100 µg/mL), chloramphenicol (15 µg/mL), kanamycin (50 µg/mL), respectively.

### Construction of LT2 Mutant and FLAG-tagged Strains

The *Δfis* strain was constructed by substitution of *fis* with a chloramphenicol acetyltransferase gene (*cat*) using the phage lambda Red recombination system [40]. The *fis*-FLAG strain was constructed by substitution of the *fis* termination codon with the 3×FLAG epitope and a chloramphenicol resistance cassette amplified from the plasmid pLW1600F [41] using the same recombination system.

The *ompR* mutant strains were generated by substitution of *ompR* with a kanamycin resistance cassette using the Red recombination system in LT2 and *Δfis*. For the overexpression of *ompR*, the *ompR* PCR product was digested with the restriction enzymes *Eco*RI and *Bam*HI, and ligated into a low copy plasmid pwsk129 [42]. The plasmid pLW1599 containing the cloned *ompR* gene was then transferred into *Δfis* [42].

Deletion of Fis-binding sites in genes *invE*, *invC* and *spaO* in LT2 were made by substitutions of the corresponding sites with a kanamycin resistance gene (*kan*) using the Red recombination system. The relevant controls were constructed by the insertion of *kan* upstream of the Fis-binding sites on genes *invE*, *invC* and *spaO,* respectively in LT2. Deletion of genes *flhD, fruR, fucR, gutM, pocR* and *prpR* in LT2 wild-type and *Δfis* were generated, respectively, by substitutions of the corresponding sites with *kan* using the Red recombination system. All primers designed for deletion and verification tests are shown in Table S2.

### ChIP

The *S*. *enterica* LT2 *fis*-FLAG strain was used to perform all ChIP-seq experiments. Cells were grown aerobically at 37°C to mid-exponential (OD A_600_ =  approximately 0.6) phases. Formaldehyde was then added to a final concentration of 1%. After 25 min of incubation at room temperature, 0.5 M glycine was added for further 5 min to quench the unused formaldehyde. Cross-linked cells were harvested and washed three times with ice-cold Tris-buffered saline (TBS). Cells were resuspended in 1 mL of lysis buffer composed of 50 mM Tris-HCl (pH 7.5), 100 mM NaCl, 1 mM EDTA, 1 mM protease inhibitor cocktail (Sigma-Aldrich), 20 mg/mL lysozyme and 0.1 mg/mL RNase A. The cells were incubated for 30 min at 37°C and 1 mL 2×immunoprecipitation (IP) buffer (100 mM Tris-HCl (pH 7.5), 200 mM NaCl, 1 mM EDTA, 2% (v/v) Triton X-100) was added. The lysate was then sonicated (Hielscher) to an average size of approximately 250 bp with 20 cycles of 30 s on/off at 95% amplitude. Insoluble cell debris was removed by centrifugation at 22,000 RCF for 10 min at 4°C, and the supernatant was split into two 900 µL aliquots. The remaining 200 µL was kept to check the size of the DNA fragments.

Each 900 µL aliquot was incubated with 30 µL Dynabeads Protein A (Invitrogen) on a rotary shaker for 1 h at room temperature to remove non-specifically binding complexes. The supernatant was then collected and incubated with 50 µL Protein A, pre-diluted with PBST (PBS buffer at pH 7.4, 0.02% Tween 20), as mock-IP sample. The IP sample was added with FLAG mouse monoclonal antibody (Sigma-Aldrich) in the supernatant. Both samples were incubated on a rotary shaker at 4°C for 4 h, and washed once with IP buffer, once with IP buffer +500 mM NaCl, once with wash buffer (10 mM Tris-HCl buffer at pH 8.0, 250 mM LiCl, 1%[v/v] Triton X-100, and 1 mM EDTA), and once with TE buffer (pH 7.5) in order. After removing the TE buffer, the beads were resuspended in 200 µL elution buffer (50 mM Tris-HCl buffer at pH 8.0, 10 mM EDTA, and 1% SDS) and eluted at 65°C for 20 min. DNA was purified and recovered by standard phenol-chloroform extraction and ethanol precipitation with 5 mg of glycogen (Invitrogen).

### RNA Extraction

To prepare cells for RNA extraction, 100 mL of fresh LB was inoculated from an overnight culture (1∶200) and incubated at 180 rpm at 37°C. *S. enterica* LT2 and the *Δfis* were collected at mid-exponential phase (OD_600_ = 0.6). RNA was extracted using TRIzol Reagent (Invitrogen) according to the manufacturer’s protocol. RNA samples were further purified using the RNeasy Mini Kit (Invitrogen). The bacterial 23S and 16S rRNA was then depleted using the MicrobExpress Kit (Invitrogen). RNA quality was determined using a Bioanalyser (Thermo) and by visualization following 1% agarose gel electrophoresis. RNA was quantified using the NanoDrop-2000 after every manipulation step.

### Library Construction and Solexa Sequencing

Library construction of immunoprecipitated DNA samples was carried out using the Next DNA Sample Prep Master Mix Set 1 Kit (NEB) following the manufacturer’s instructions. DNA samples were purified using the QIA quick PCR Purification Kit (QIAGEN) and the QIA quick Gel Extraction Kit (QIAGEN) after each manipulation step. Samples were loaded at a concentration of 8 pM.

RNA library construction was carried out using the mRNA-Seq 8-Sample Prep Kit (Illumina) according to the manufacturer’s protocol. Samples were loaded at a concentration of 10 pM.

### RT-PCR for ChIP-seq and RNA-seq Validation

To measure the enrichment of the Fis-binding targets in the immunoprecipitated DNA samples, RT-PCR was performed using the 7300 Fast Real-Time PCR systems (Applied Biosystems). IP or mock-IP DNA (1 µL) was used as a template, and the amplifications were performed using specific primers (Table S2) and SYBR mix (QIAGEN). To measure gene transcription in different strains, RT-PCR was carried out using specific primers based on targeted genes. Total RNA (1.0 µg) was reverse transcribed to generate cDNA as the template for RT-PCR. The RT-PCR conditions were as follows: 25 µL SYBR mix (QIAGEN), 1 µL each primer (10 pM), 1 µL cDNA or DNA, and 22 µL ddH_2_O. Three independent technical replicates were carried out for each reaction.

### HeLa Cell Infection Assays

Infection assays using human HeLa epithelial cells (ATCC CCL-2) were performed as described previously [43]. HeLa cells (1×10^5^/well) were infected (multiplicity of infection (moi) of 10) for 30 min with bacteria grown to early exponential phase. To increase contact between the bacteria and cells, the 6-well plates were centrifuged at 1000×*g* for 5 min, and incubated for 40 min at 37°C in 5% CO_2_. Macrophages were washed three times in PBS to remove non-invasive bacterial cells, and fresh RPMI-1640 medium containing 50 µg/mL gentamicin was added to kill remaining extracellular bacteria. After 1 h, the rate of invasion was calculated according to the number of recovered bacterial cells relative to the input number. Experiments were carried out in triplicate.

### Macrophage Infection Assays

Infection assays using murine RAW 264.7 macrophage cell (ATCC TIB-71) were performed as previously described [43]. RAW264.7 macrophages were incubated in the RPMI-1640 medium and seeded (1×10^6^ cells/well) in 6-well plates one day prior to infection. Bacteria were harvested at the exponential phase and used for infection of RAW264.7 cells (moi, 10∶1). Bacterial cells were centrifuged (37°C, 800×*g*, 5 min) onto the macrophages and incubated for 40 min at 37°C in 5% CO_2_. Macrophages were washed three times with PBS to remove non-invasive bacterial cells; this h was defined as the 0 h time-point. After washing, fresh RPMI-1640 medium containing 50 µg/mL gentamicin was added to kill remaining non-invasive bacterial cells. After 1 h, the medium was replaced with RPMI-1640 medium containing 15 µg/mL gentamicin, and incubated for an additional 28 h. The number of intracellular bacteria was determined at 0, 1, 2, 4, 6, 8, 12, 21, 24 and 28 h. To estimate the amount of intracellular bacteria at each time point, cells were lysed using 0.1% SDS, and cell lysates were collected and serially diluted 10-fold in PBS, and aliquots were plated onto LB agar to enumerate bacterial colony-forming units (cfu) [44]. Experiments were carried out in triplicate.

### Fis Purification

Fis protein was purified by glutathione sepharose high performance (GE Healthcare) according to the manufacture’s protocol. The amplified *fis* product was cloned into pGEX4T-1 to generate the plasmid pLW1601, and then transformed into *E. coli* BL21 to generate strain H2114. Strain H2114 was grown at 37°C to OD_600_ = 0.4, and Fis expression was induced by the addition of 0.1 mM isopropyl-β-D-thiogalactopyranoside (IPTG) for 3 h at 30°C. Fis purity was assessed by Coomassie stained SDS-polyacrylamide gel electrophoresis (PAGE), and its concentration was quantified by Bradford assay.

### Electrophoretic Mobility Shift Assay

Gel mobility shift assays were performed by incubating amplified Fis-binding DNA fragments (1 nM) at 25°C for 20 min with various concentrations of Fis protein (0–400 nM) in a 20 µL solution containing 20 mM Tris-HCl (pH 7.5), 80 mM NaCl, 0.1 mM EDTA and 1 mM DTT. The boundaries of the Fis-binding DNA found within intergenic regions or the open reading frame (ORF) regions were shown in Table S3. DNA fragments containing the *dmsA* ORF region and the *ompR* promoter region were PCR amplified as negative and positive controls, respectively [4], [39]. Samples were loaded with native binding buffer on a 6.0% polyacrylamide gel in 0.5×TBE. Gel staining was operated according to the manufacture’s protocol [45].

### Analysis of Fis-binding Regions from ChIP-seq Data

To identify Fis-binding regions on the LT2 chromosome, we mapped ChIP sequences to the genome. The sequencing reads were mapped to both strands and the distribution of read counts for each basepair formed a standard plot that range between 0–200 at the genomic scale. An in-house perl script algorithm, coupled with the RPKM (reads per kilobase per million mapped reads) value [46], was used to detect binding peaks. Several parameters were set up, including: μ = 20 (if the plot value of a base pair reached 20, the base pair was considered to be the start site of a potential binding region); ρ = 100 (a potential binding region was determined as a real binding region only if the plot value of the peak in this region reached 100). After identification of the potential Fis-binding regions corresponding to these conditions, RPKM values were re-calculated. False positive binding regions (RPKM in the mock-IP greater than those in the IP) were removed. All the remaining Fis-binding regions were then considered to be effective Fis-binding targets.

### Analysis of Fis-regulated Genes from RNA-seq Data

All the raw FastQ files were cleaned by SolexQA [47]. To obtain estimates of transcription levels, TopHat (v1.3.1) [48] was used to align the trimmed sequencing reads with the LT2 genome. The genome and gene annotation used in this study was obtained from the NCBI website. Cufflinks (v1.1.0) [49] was then used to estimate gene transcription levels based on the same gene model annotations. To select the genes with significant differential expression, the Cufflinks output was parsed by a perl script. To present the gene expression levels, RPKM was used as normalized metrics. RPKM values [46] were determined for all genes in each of the samples tested. In our research, genes showing greater than a twofold change (ratio of RPKM) in transcription between cells grown in the presence and absence of the *fis* gene were identified.

### Motif-searching

To identify Fis-binding motifs, the sequences of Fis-binding regions obtained from ChIP-seq were analyzed using MEME-ChIP software [50]–[52], with the following parameters: zero or one motif per sequence; motif width ranging from 6–20; searching both strands of the sequences; using a background distribution file containing the mono-nucleotide frequencies of the LT2 chromosome. The motif with the lowest E-value was considered as the significant motif.

## Results

### Genome-wide Mapping of Fis-binding Sites

**Figure 1:**
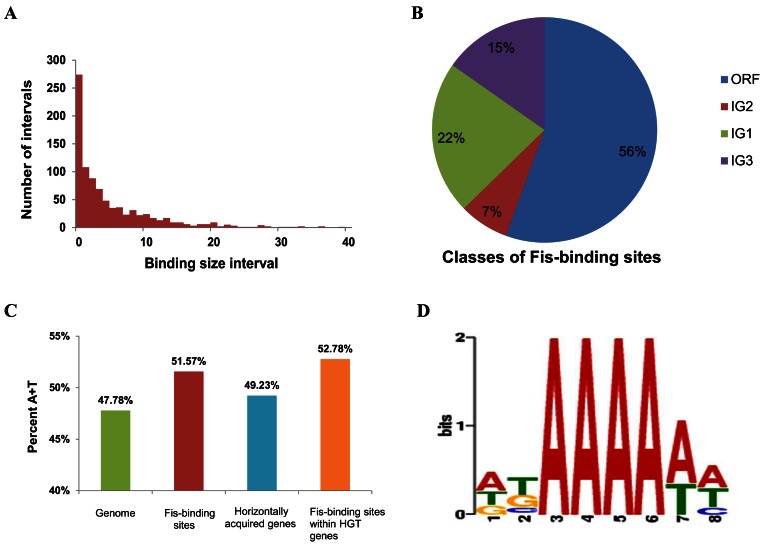
Properties of Fis-binding regions. **a**, A histogram of the lengths of the intervals of Fis-binding sites. **b**, Distribution of Fis-binding sites in the four classes. **c**, Average A+T contents of various classes of genes. **d**, The most significant motif found in Fis-binding regions. The height of each individual symbol reflects its prevalence at a given position, and the height of each column is proportional to the positional information content in this position.

Both mock-IP and IP samples of *S. enterica* serovar Typhimurium strain LT2 were collected in the mid-exponential phase (OD_600_ = 0.6), and ChIP-seq analysis was performed. For mock-IP samples, 6,576,604 reads of 100-nt length were mapped to the LT2 genome, amounting to 120-fold coverage; for IP samples, 3,350,972 reads were mapped to the genome, amounting to 50-fold coverage (Table 1). After removing non-specific binding regions for which read counts from mock-IP samples are greater than those from IP samples, a total of 885 binding regions in 943 genes (Table S3) were detected. Fis was found to bind to a total of 309 kb (approximately 8%) sequences on the LT2 genome. The average length of Fis-binding regions is estimated to be 349 bp, and the average length of Fis-binding region intervals is 5.21 kb (Figure 1A). Validation of the ChIP-seq results was performed by RT-PCR for 22 genes, including 20 Fis-binding genes and two genes (*carA* and *hupA*) not bound by Fis as controls. The 20 genes included six previously identified Fis-binding genes (*sodC*, *hin*, *nrfA*, *rrsA*, *tgt*, *dps*) [4], and 14 genes newly identified as Fis-binding genes in this study (STM0212, STM1250, STM2332, *sopB, ptsG, sscB, ssaU, sipB, sipC, invC, accC, ompR, mgtC* and *metJ*). All six previously identified and 14 newly detected Fis-binding regions exhibited enrichment as a log_2_ ratio range of 0.47 to 22.76, and the two control regions showed no significant enrichment (Table S4). These results indicate that the majority of Fis-binding regions identified by ChIP-seq are reliable.

**Table 1:** Summary of the ChIP-seq and RNA-seq data.

−	ChIP-seq		RNA-seq	
Sample	Mock-IP	IP	LT2	*Δfis*
Number of reads	6,576,604	3,350,972	13,894,928	14,708,050
Number of mappable reads	5,717,748	2,017,349	13,202,514	14,089,439
Number of reads per base	118	42	272	290
Number of binding regions^a^	−	885 bp	−	−
Average length ofbinding sites	−	349 bp	−	−

a The number of Fis binding regions.

The 885 Fis-binding regions can be classified into four categories according to the relative position between binding regions and related genes: ORF, IG1, IG2 and IG3. The ORF category consists of Fis-binding peaks found within ORFs regions. Among the 885 binding regions, 492 (55.59%) regions belongs to the ORF category. The IG1 contains those found in intergenic regions between two genes transcribed in the same direction; 195 (22.03%) regions were classified as IG1. The IG2 category consists of Fis-binding peaks found in intergenic regions between two divergently transcribed genes; 63 (7.12%) regions of this category were detected. All of the remaining binding regions (135, 15.25%) were classified as IG3, which are partly located in intergenic region and partly within the ORF (Figure 1B).

The average A+T content of the Fis-binding sites was estimated to be 51.57% (Figure 1C), which is higher than that of the LT2 chromosome (47.78%) [24]. LT2 contains 937 horizontally acquired genes [24], and 207 of the 885 Fis-binding regions are within horizontally acquired genes. The average A+T content of the Fis-binding sites in horizontally acquired genes is 52.78%, higher than that of total horizontally acquired genes (49.23%). These data suggest that Fis binds preferentially to regions of higher A+T content in LT2, which is in accordance with that found in *E. coli* K12 [3], [4], [16], [53].

The unbiased motif-searching algorithm MEME was used to identify the most significant Fis-binding DNA sequence motif in LT2. The motif with the lowest E-value (5.2e-039) was selected (8 nt in length), the log likelihood ratio of the motif being 1749 with an information content of 10.6 bits (Figure 1D). The motif is non-palindromic, consisting mainly of A/T nucleotides, with four consecutive A nucleotides in the center. The motif is present in 641 of the 885 Fis-binding regions and appears 1592 times in total, twice per binding region on average.

### Fis Regulation on Global Gene Transcription

**Figure 2:**
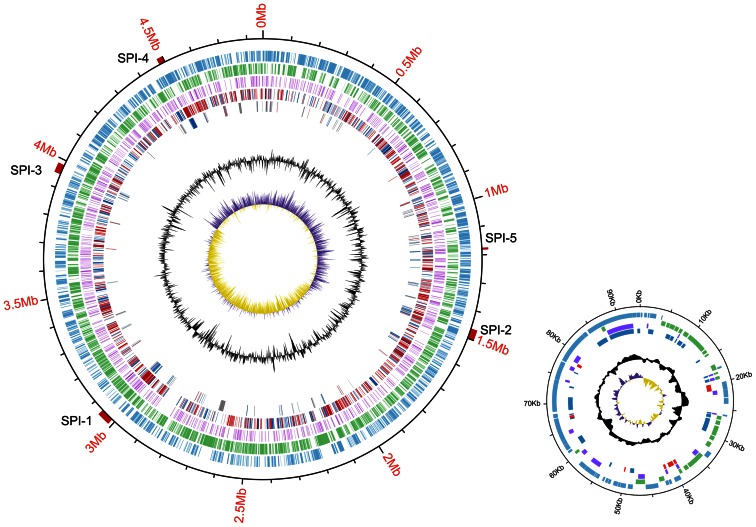
Correlation between ChIP-seq and transcriptomic analyses of the *S*. *enterica* LT2 chromosome and plasmid. The outer ring (ring 1) shows the location relative to position zero measured in millions of base-pairs (Mbp) of the *S*. *enterica* LT2 genome. Ring 2 shows the positive strand, and ring 3 shows the negative strand. The Fis-binding regions are shown in ring 4. Ring 5 shows changes in gene expression in *Δfis* compared to the parental LT2 strain. Genes that are down-regulated in *Δfis* are shown in red; genes that are up-regulated are shown in blue. Ring 6 shows Fis-binding genes that are HGT genes: red indicates genes that are down-regulated in *Δfis*, blue indicates genes that are up-regulated, gray indicates genes that are not regulated by Fis. Ring 7 shows the GC%, and ring 8 indicates the GC skew [81] (purple and yellow regions have a GC skew that is less than or greater than the genomic average, respectively). The location of the SPI 1–5 is indicated.

Fis regulation at the global level in LT2 in the mid-exponential phase was studied by RNA-seq on the wild-type and *Δfis* (Figure 2). The cDNA reads obtained for the wild-type and *Δfis* were 13,202,514 and 14,089,439, with the map-rate of 95% and 96%, respectively (Table 1). A total of 1646 genes were found to be differently transcribed between the wild-type and *Δfis*; 657 and 989 genes exhibiting higher or lower levels of transcription, respectively, in *Δfis* (Table S5). The RNA-seq results were confirmed by performing RT-PCR analysis of the wild-type and *Δfis* under the same culture condition as RNA-seq experiments. RT-PCR analysis targeted 12 genes, including five Fis-independent genes and seven Fis-dependent genes, and the results corresponded well with the RNA-seq data (Table S6).

**Figure 3:**
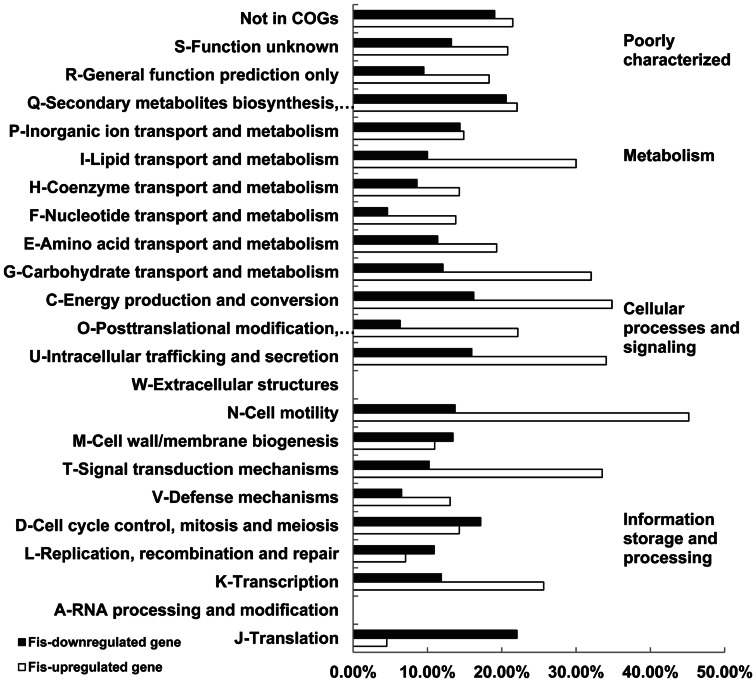
Clusters of orthologous groups (COG) analysis of Fis-regulated genes in *S. enterica* LT2. COG categories are indicated by the figures on the right and sub-categories are listed on the left. The x-axis represents the percentage of genes in the corresponding class. Genes which are activated and repressed by Fis are indicated in white and black, respectively.

RNA-seq results indicated that Fis prefers to positively influence gene transcription in LT2 in the mid-exponential phase. The genes differentially transcribed were classified into functional categories based on clusters of orthologous groups (COG) designations (www.ncbi.nlm.nih.gov/COG), and the percentage of up-regulated and down-regulated genes in *Δfis* in each COG category was calculated (Figure 3). Fis-regulated genes fell within almost all COGs except for genes involved in extracellular structures, RNA processing and modification. Among 23 COGs, 17 contained more genes activated by Fis than those repressed by Fis. The COG with the largest proportion of Fis-activated genes was the cell motility class of which approximately 45% genes were up-regulated and 13% genes were down-regulated by Fis. In the other four COGs, there were more genes repressed by Fis than those activated by Fis. The COG with lowest proportion of Fis-activated genes was the translation class, of which 22% genes were Fis-repressed genes, and 4% were Fis-activated genes (Figure 3).

Among the 1646 differently transcribed genes between the wild-type and *Δfis*, 317 were Fis-binding genes, which included 207 genes (65.30%) with lower transcription in *Δfis*, and 110 (34.70%) genes with higher transcription (Table 2). It was also found that the activated gene ratio for Fis-binding genes (65.30%) was higher than that for all Fis-regulated genes (60.09%). These results indicated that, in LT2, Fis tends to perform an indirect regulatory role, while its direct regulatory role was shown to involve preferential up-regulation of the Fis-binding genes.

**Table 2:** The number of genes regulated by Fis.

	Activation	Repression	Total	Silent^c^	Total
Direct^a^	207	110	317	626	943
Indirect^b^	782	557	1329	−	−
Total	989	657	1646	−	−

a Genes which are both regulated and bound by Fis.

b Genes which are regulated by Fis but are not associated with Fis binding.

c Genes which are Fis-binding genes but are not regulated by Fis.

Of the 1646 Fis-regulated genes, 1329 were not associated with Fis-binding (Table S3 and S5). Of these 1329 genes, 419 are known to be regulated by 55 transcription factors (TFs) in LT2 (Regulon DB), including 27 Fis-independent and 28 Fis-dependent transcription factors (TFs). It is known that the 28 Fis-dependent TFs regulate 303 of those 419 genes (Regulon DB); thus, it is highly likely that Fis controls the transcription of these 303 genes by regulating corresponding TFs. We randomly selected five of the 28 TF genes (*fruR, fucR, flhD, gutM* and *prpR*) [54]–[58] to perform gene knockout experiments, and the transcription of 19 genes regulated by these five TFs were compared by RT-PCR between the wild-type and *Δfis*, and between *Δ*TF and *Δ*TF*Δfis*. Three of the 19 genes showed 3.22 to 50.00-fold increases and 16 genes showed 1.25 to 26.35- fold reductions in transcript levels in *Δfis* compared to the wild-type. However, in a TF mutant background, the three Fis-repressed genes exhibited only 1.06 to 5.00-fold higher transcription caused by the mutation of *fis*, and the effect of Fis on the 16 Fis-activated genes was attenuated or non-existent (0.27 to 7.67-fold) (Table 3). This confirmed that Fis controls the transcription of these 19 genes by regulating the five TFs.

**Table 3:** TF genes effect on Fis-regulated genes.

TF	TF regulated genes	wt/*Δfis* a	*ΔTF*/*ΔTFΔfis* b
FruR	*fruK*	0.02	0.94
	*eno*	0.03	0.20
	*cydA*	1.25	1.10
	*marR*	0.31	0.40
FucR	*fucI*	2.45	0.35
	*fucK*	2.64	0.37
	*fucO*	2.20	0.27
FlhD	*flgD*	5.70	1.36
	*flgE*	5.03	0.55
	*flgF*	4.72	0.70
	*flgG*	4.44	0.76
	*flgC*	4.82	0.81
GutM	*srlA*	26.35	7.67
	*srlD*	6.45	1.13
	*srlR*	5.21	1.64
PrpR	*prpB*	6.45	3.12
	*prpC*	3.68	2.66
	*prpD*	2.89	0.73
	*prpE*	3.46	1.55

a Ratio between LT2 wild-type and *Δfis* obtained from RT-PCR.

b Ratio between *ΔTF* and *ΔTF/ΔTFΔfis* obtained from RT-PCR.

### Fis Regulates B_12_ Biosynthesis Genes by Binding to and Activating PocR

B_12_ is a known cofactor for numerous enzymes mediating methylation, reduction, and intramolecular rearrangements [59]–[61]. B_12_ biosynthesis genes, which are not present in *E. coli,* were acquired by horizontal gene transfer (HGT) in *Salmonella* [62]. In LT2, a total of 30 genes are required for B_12_ biosynthesis, and 25 of these are clustered in the *cob* operon [61]. The transcription of the *cob* operon is mainly controlled by a trans-acting protein encoded by the *pocR* gene, which is located upstream of the *cob* operon [61], [63].

**Figure 4:**
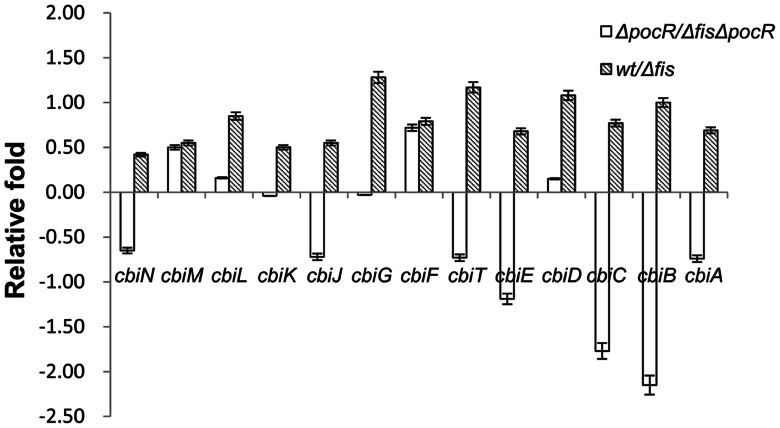
Effect of *pocR* on B_12_ genes. The *y*-axis represents the relative fold enrichments (log_2_ of expression ratio) between the LT2 wild-type (*wt*) and *Δfis* with or without the *pocR* deletion. If the number of relative fold is greater than zero, it represents the expression level of genes in the LT2 wild-type (*ΔpocR*) is higher than that in *Δfis* (*ΔfisΔpocR*); if the number of relative fold is less than zero, it represents the expression level of genes in the LT2 wild-type (*ΔpocR*) is lower than that in *Δfis* (*ΔfisΔpocR*). Each bar represents the statistical mean from three independent biological replicates.

In this study, none of the 25 *cob* genes were found to be associated with Fis-binding (Table S3), yet 16 of them were down-regulated upon deletion of *fis,* indicating that Fis up–regulates these genes (Table S5). The *pocR* gene was found to be activated 5.27-fold by Fis and a Fis-binding site was identified within the gene (Table S3 and S5). Evaluating of the transcriptional differences in the 16 *cob* genes in the wild-type, *Δfis*, *ΔpocR* and *ΔfisΔpocR* by RT-PCR revealed 1.34 to 2.43-fold lower expression of these 16 *cob* genes in *Δfis* compared to the wild-type (Table S7). Four of the 16 genes showed 1.11 to 1.65-fold decreases, and nine of those 16 genes exhibited 1.03 to 4.44-fold increases in transcription in *ΔfisΔpocR* comparing to *ΔpocR* (Figure 4), indicating that the positive effect of Fis on these 13 genes is significantly attenuated or absent (Figure 4) due to the deletion of *pocR*. The reason that the expression of *cob* genes in *pocR* deleted strains is still dependent of Fis effect is Fis may also control the expression of B_12_ biosynthesis genes through other unknown negative regulators. In addition, the other three of the 16 genes (*cbiH*, *cbiQ* and *cbiO*), showed 1.56, 1.77, 1.34-fold decreases in transcription, respectively, in *ΔfisΔpocR* compared to the *ΔpocR*, which is similar to the expression changes (1.55, 1.52, 1.22-fold) of those genes between *Δfis* and the wild-type. The result showed that Fis activates the transcription of B_12_ biosynthesis genes mainly through controlling the expression of *pocR*.

### Fis Effect on LT2 Invasion and Fis Regulation on SPI Genes

**Figure 5:**
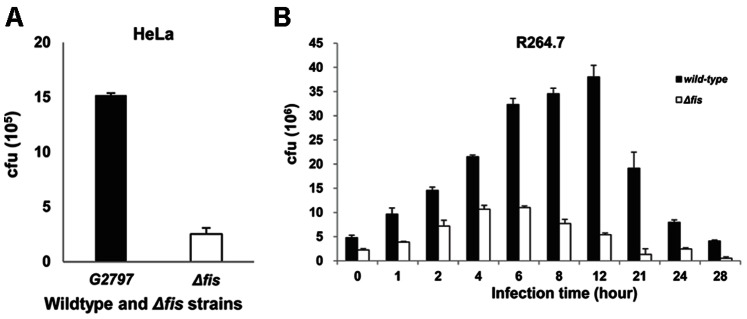
Fis effect on invasion and intracellular replication of LT2 within cells. **a**, HeLa epithelial cell infection assays: the data show the number of bacteria detected one hour after invasion of HeLa cells. Wild-type and *Δfis* indicate the LT2 wild-type and *Δfis.* **b**, Macrophage infection assays using murine RAW 264.7 macrophage cells: increased intracellular replication of the LT2 wild-type and *Δfis* strains during the infection. The *y*-axis represents the number of intracellular bacteria in macrophages at 0, 1, 2, 4, 6, 8, 12, 21, 24 and 28 hours post-infection. Each bar represents the statistical mean from three independent biological replicates.

The invasion ability of *S. enterica* is dependent on its ability to invade intestinal epithelial cells and to survive inside macrophage cells [43], [64], [65]. In a previous study, *S. enterica* SL1344 *Δfis* exhibited 50 to 100-fold decreased ability to invade HEp-2 (epithelial) cell, compared to the wild-type strain [30]. In this study, the effect of Fis on the invasion of HeLa cells by LT2 was evaluated, and LT2 *Δfis* also exhibited decreased ability (5.02-fold) (Figure 5A).

We also analyzed the effect of Fis on the survival of LT2 inside murine macrophage cells. The number of *Δfis* within cells was 2.00 to 14.42-fold less than within the wild-type strain at each time-point from 0 h to 28 h post-infection (Figure 4B). These results indicated that Fis enhances invasion and intracellular replication ability of LT2 within the host cell. It is well known that SPI-1 and SPI-2 genes are mainly responsible for the invasion and intracellular replication of LT2 within host cells; thus, we further investigated the effect of Fis on the expression of SPI-1 and SPI-2 genes. In comparisons of the expression profiles of LT2 wild-type and *Δfis,* 63 of the 94 SPI-1 and SPI-2 genes were found to be regulated by Fis, including 55 Fis up-regulated genes and eight Fis down-regulated genes (Figure S1).

Of the 63 Fis-regulated genes (Table S8), only 23 could be directly bound by Fis in ChIP-seq analysis, indicating that these genes are directly regulated by Fis. We then randomly selected 16 genes, including *hilC, sipA*, *sipB*, *spaS*, *sicA*, *invE*, *invC, spaO* of SPI-1, and *orf242, ssrA,* s*saB, sscA, sscB, sseC, sseF, ssaV* of SPI-2, to perform gel mobility shift assays. As expected, the result showed that when the concentration of Fis protein (0–400 nM) was increased, more Fis-DNA complex and less free DNA were detected for all 16 genes (Figure S2). This confirmed the binding of Fis to these 16 genes.

**Figure 6:**
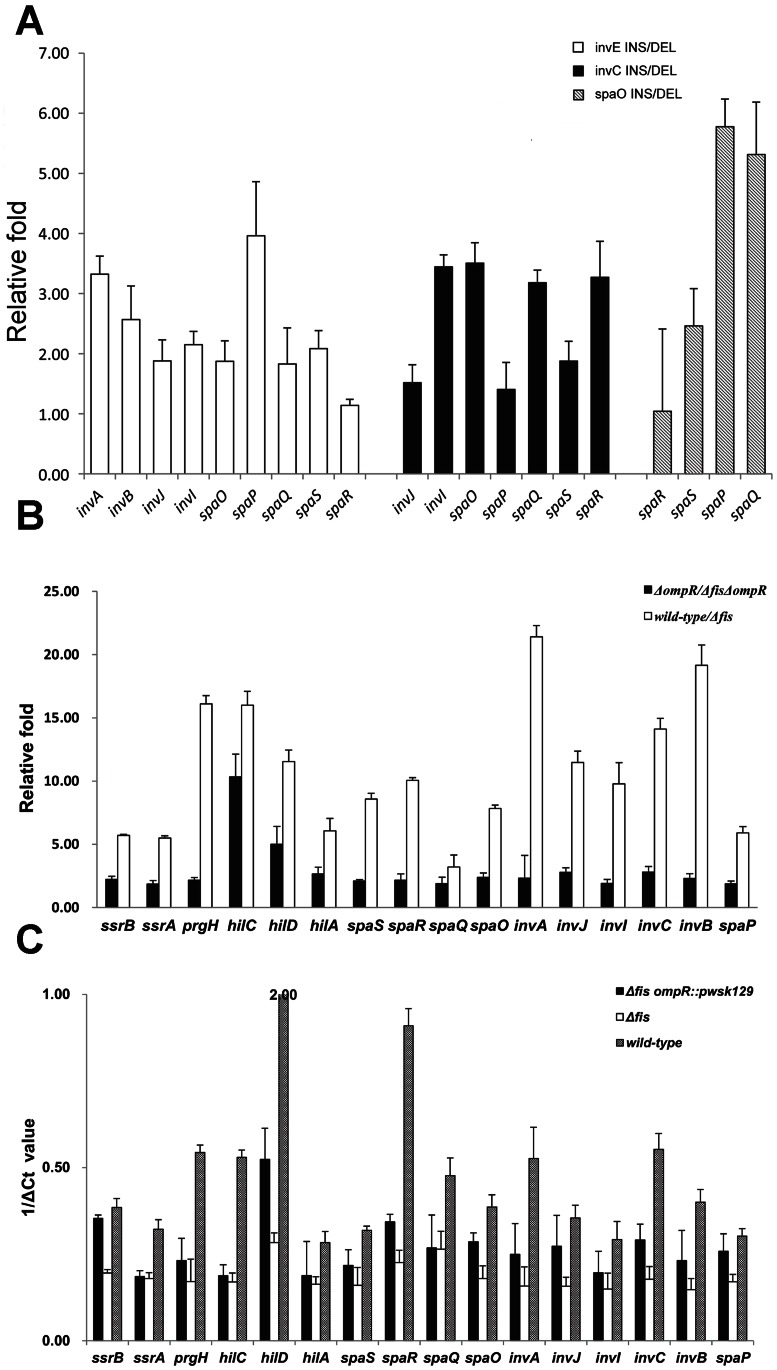
Effect of OmpR and Fis-binding sites on SPI. **a**, The *y*-axis represents changes in the transcription of genes downstream of *invE, invC, spaO genes* without corresponding upstream binding sites. The binding sites in *invE, invC, spaO* are replaced by *kan*, and indicated by DEL. INS represents strains with insertion of a *kan* gene upstream of the Fis-binding sites in the *invE, invC, spaO* genes. **b**, Analysis of the effect of Fis on SPI genes through *ompR*. Wild-type and *Δfis* indicate the LT2 wild-type and *Δfis.* The *y*-axis represents the relative fold enrichments between the LT2 wild-type and *Δfis* with or without the *ompR* deletion. **c**, The 1/ΔCt value of the RT-PCR results. Larger values represent higher gene transcription. Each bar represents the statistical mean from three independent biological replicates.

We also found that eight Fis-activated genes, although not associated with Fis-binding, were proximally located downstream of three Fis-binding genes (*invE*, *invC*, and *spaO*). These included *InvA* and *invB* located downstream of *invE*, *invI* and *invJ* located downstream of *invC*, and *spaP, spaQ, spaR* and *spaS* located downstream of *spaO*. In *Δfis*, all eight genes showed greater than 23.37-fold decrease in transcription. The Fis-binding sites in the ORF regions of *invE*, *invC*, and *spaO* were substituted by *kan* in the wild-type. As a control, *kan* was also inserted upstream of the binding sites in *invE*, *invC*, and *spaO* in the wild-type. RT-PCR assays showed that deletion of the corresponding Fis-binding sites led to 1.13 to 5.78-fold transcriptional decrease of these genes compared with corresponding control strains (Figure 6A). These results indicated that Fis regulates not only genes to which it binds directly, but also genes downstream of the Fis-binding genes (Table S8).

The other 21 SPI genes, which are not associated with Fis binding, are probably regulated by Fis through control of the expression of global regulator or SPI regulators (Table S8). Among the SPI regulators, only *hilC* and *ssrA* were both Fis-regulated and Fis-binding genes, indicating that they are regulated by Fis directly. HilC plays a key role in co-ordinating expression of the SPI-1 genes [66]–[68], and its transcription was found to be decreased 43.82-fold in *Δfis*. Eleven Fis-regulated SPI genes have been reported to be under the control of HilC [25], [32]. SsrA-SsrB is a two-component regulatory system for SPI-2, which includes SsrA as the predicted integral membrane cognate sensor and SsrB as the response regulator binding to the promoters of all SPI-2 functional gene clusters [25], [69]. The transcription of *ssrA* was found to decrease 2.90-fold in *Δfis*, and 10 Fis-regulated SPI genes have been reported to be under the control of SsrA [25], [70].

Another SPI regulator, HilD, which is not associated with Fis-binding, acts in an ordered fashion with HilC to coordinately activate expression of the SPI-1 genes. The transcription of *hilD* was also found to be decreased 10.03-fold in *Δfis*. However, *hilD* were not found to be bound by Fis. We proposed that *hilD* was probably indirectly regulated by Fis through other proteins. The *barA, fur* and *ompR* genes were reported to be positively regulators of *hilD* [32], [39], [71], and in this study, they were all found to be associated with Fis binding (Table S3). However, *Δfis*, *barA* and *fur* were found to maintain their transcription level, and only the expression of *ompR* decreased 2.41-fold. OmpR is associated with Fis binding (Table S5), which is consistent with previous study [39], indicating that *ompR* is regulated by Fis directly. OmpR was also reported to bind to the promoter of the SPI-2 gene regulator *ssrAB* [69], [72]. In this study, the transcription of 14 SPI-1 and 2 SPI-2 genes was evaluated by RT-PCR in the wild-type, *Δfis*, *ΔompR* and *ΔfisΔompR*. Obvious decrease (3.20 to 21.41-fold) in the transcription levels of these genes were observed in *Δfis* compared to the wild-type (Figure 6B). However, this decrease was less marked (1.85 to10.34-fold) in the *ΔfisΔompR* strain compared to the *ΔompR* strain. We then overexpressed *ompR* in *Δfis*, and found that the increased transcription of the SPI-1 and SPI-2 genes was recovered (1.04 to 6.36-fold increase), thus partly compensating for the effect caused by the *fis* deletion (Figure 6C). These results suggest that Fis positively influences the SPI-1 and SPI-2 regulators by binding to and activating *ompR*.

**Figure 7:**
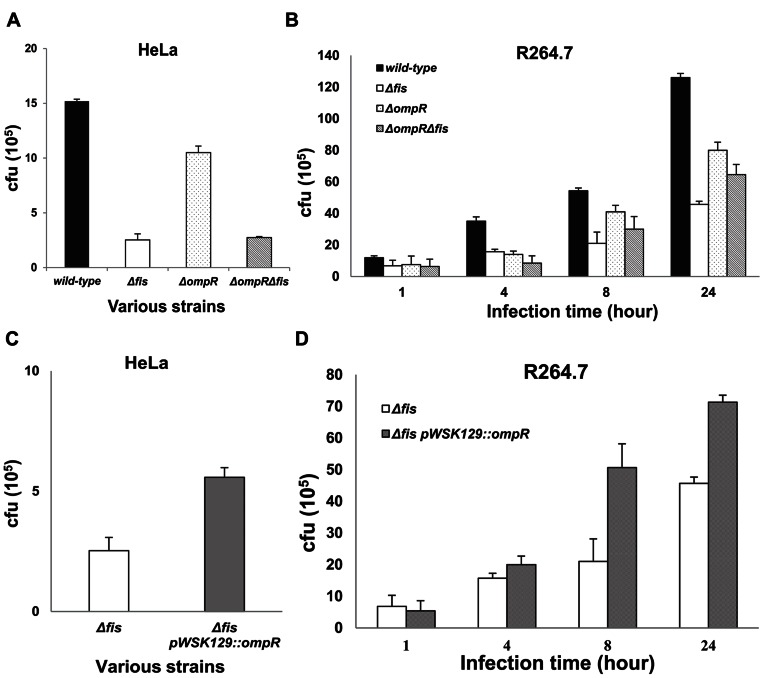
OmpR effect on invasion and intracellular replication of LT2 within cells. **a**, HeLa epithelial cell infection assays: the data show the number of bacteria detected one hour after invasion of HeLa cells. **b**, Macrophage infection assay using murine RAW 264.7 macrophage cells: increased intracellular replication of the *S. enterica* LT2 wild-type, *Δfis*, *ΔompR*, *ΔfisΔompR* during infection of RAW264.7 macrophages. The data show the number of viable intracellular bacteria in macrophages at 1, 4, 8 and 24 hours post-infection. **c**, *S*. *enterica* LT2 *Δfis* and *Δfis* pwsk129::*ompR* infection of HeLa cells: the data show the number of bacteria detected one hour after invasion of HeLa cells. **d,** *S*. *enterica* LT2 *Δfis* and *Δfis* pwsk129::*ompR* infect of RAW264.7 macrophage cells. The data show the number of viable bacteria in macrophages at 1, 4, 8 and 24 hours post-infection. Each bar represents the statistical mean from three independent biological replicates.

To confirm the role of *ompR* in the effect of Fis on LT2 infection, cell invasion assays were carried out. The discrepancy in the HeLa invasion rate observed between the *ΔompR* and *ΔfisΔompR* (3.83-fold decrease in *ΔfisΔompR*) was found to be smaller than that between the wild-type and *Δfis* (6.01-fold decrease in *Δfis*) (Figure 7A). Similar results were also detected in the infection assay using murine RAW 264.7 macrophages (Figure 7B). Smaller discrepancies were detected for *ompR* deletion-mutant strains at 0 to 24 h post-infection (1.18 to 1.64-fold decrease in *ΔfisΔompR*). Moreover, overexpression of *ompR* also increased the invasion (2.21-fold) and survival ability of *Δfis* (1.27 to 2.41-fold) (Figure 7C and 7D). These results indicated that *ompR* plays an important role in Fis regulation of SPI-1 and SPI-2 genes, and influences the infection and replication of LT2 in host cells.

## Discussion

By using high-throughput sequencing methods, we clarified the genome-wide distribution of binding regions and global regulation pattern of Fis, one of the most important nucleoid-associated regulators, in *S. enterica* serovar Typhimurium LT2. Furthermore, by using cell infection assay, we showed that Fis not only enhances invasion ability, but also intracellular replication ability of LT2 within macrophage cells. Most importantly, Fis was found to activate SPI genes, which are essential for the virulence of *S. enterica*. In this study, the three regulatory modes for Fis on SPI genes were illustrated for the first time.

We identified 885 Fis-binding sites spread over on a total of 943 genes in LT2. Compared to the reported 894 Fis-binding sites on 1341 genes in K12 [4], some new features were observed in LT2: i) There is a different global regulation pattern of Fis in LT2. Only 145 common genes are bound by Fis in both K12 and LT2, which is possibly due to the difference in their genome sequence, as 320 of the 943 Fis-binding genes in LT2 are not present in K12 [24]. This result is also consistent with the phenomenon that the DNA supercoiling levels, which are controlled by Fis, are different between *E. coli* and *S. enterica* [26]. ii) A higher percentage (23%) of Fis-binding sites was found within HGT genes in LT2, which was significantly higher than that (10%) reported in K12 [3]. The discrepancy might be due to that LT2 acquired at least 1,106 gens mainly by HGT since the divergence from *E. coli* [24]. iii) LT2 has a novel Fis-binding motif, which contains a A/T-tract (similar to that in K12), but has no conserved G/C on either side (present in K12). The two *E. coli* Fis-binding motifs [3], [4] were searched against Fis-binding sequences in LT2, but no regions of high-homology were identified.

Our RNA-seq data shows that among 1646 Fis-regulated genes in LT2, the expression of 60.09% genes are repressed in *Δfis*. In contrast, only 33.59% (310 of 923 genes) of Fis-regulated genes were found to be up-regulated in the mid-exponential phase in K12 [4]. This indicates that in LT2 Fis preferentially mediates positively regulation of genes, which is opposite from that in K12. Two hundred and thirty-six genes were found to be regulated by Fis in both K12 and LT2, we found only a small proportion (163 genes) of which are regulated by Fis with the same tendency, including genes for oxidative phosphorylation, secondary metabolism, motility and carbon utilization. Seventy-three genes were found to be differently regulated by Fis between the two strains, including genes for the energy and fatty acid metabolism, membrane transport and signal transduction (Table 4). The great differences in Fis transcriptional regulation found between LT2 and K12 may be due to the different Fis-binding sites in two strains, and the difference in culture medium and high-throughput technologies used in the two studies.

**Table 4:** KEGG pathway of genes differentially regulated by Fis in *S. enterica* LT2 and in *E. coli* K12.

Metabolism	PATH	PATH number^a^	BA^b^	BR^c^	AR^d^	RA^e^
Carbohydrate metabolism	Glycolysis	Stm00010	2	0	1	0
	Citrate cycle (TCA cycle)	Stm00020	7	0	1	0
	Pentose phosphate pathway	Stm00030	2	0	1	0
	Starch and sucrose metabolism	Stm00050	4	0	0	0
	Amino and nucleotide sugar metabolism	Stm00520	2	0	1	1
	Pyruvate metabolism	Stm00620	1	0	2	0
	Glyoxylate and dicarboxylate metabolism	Stm00630	1	0	1	0
	Propanoate metabolism	Stm00640	1	0	3	0
	Butanoate metabolism	Stm00650	3	0	2	0
	others		2	1	0	0
Energy metabolism	Oxidative phosphorylation	Stm00190	5	0	0	0
	others		4	0	6	2
Amino acid metabolism			5	1	4	0
Glycan biosynthesis and metabolism			0	2	0	0
Metabolism of cofactors and vitamins			0	2	0	0
Metabolism of terpenoids and polyketides			1	1	1	0
Biosynthesis of other secondary metabolism			4	0	1	0
Translation			1	2	0	2
Replication and repair			0	1	1	0
Membrane transport			2	1	4	0
Signal transduction			2	0	3	1
Flagellar assembly			10	0	0	0
Not in COGs			43	50	23	8
**Total**			102	61	55	41

a Pathways are numbered according to KEGG database.

b BA class includes genes which are activated by Fis both in LT2 and K12.

c BR class includes genes which are repressed by Fis both in LT2 and K12.

d AR class includes genes which are activated by Fis in LT2, but repressed in K12.

e RA class includes genes which are repressed by Fis in LT2, but activated in K12.

The effect of Fis on global transcriptional regulation in *S. enterica* Typhimurium SL1344 has been studied by Kelly *et al*. using microarrays [7], only 291 genes were found to be influenced by Fis. By comparing data of that study with our research, most of Fis-regulated genes in SL1344 were found to be consistent with those in LT2. For instance, genes involved in motility and SPI were found to be strongly down-regulated in *Δfis* in both studies. However, there are 30 Fis-regulated genes showed opposite regulation tendencies in the two studies. For instance, Kelly *et al*. reported that Fis did not affect genes involved in B_12_ production, while, our study surprisingly found that these genes were significantly repressed in *Δfis*. These differences may be due to the discrepancy in time-points analyzed in the two studies (1 hour post-subculture vs. mid-exponential phase after subculturing) and the low resolution of microarrays.

**Figure 8:**
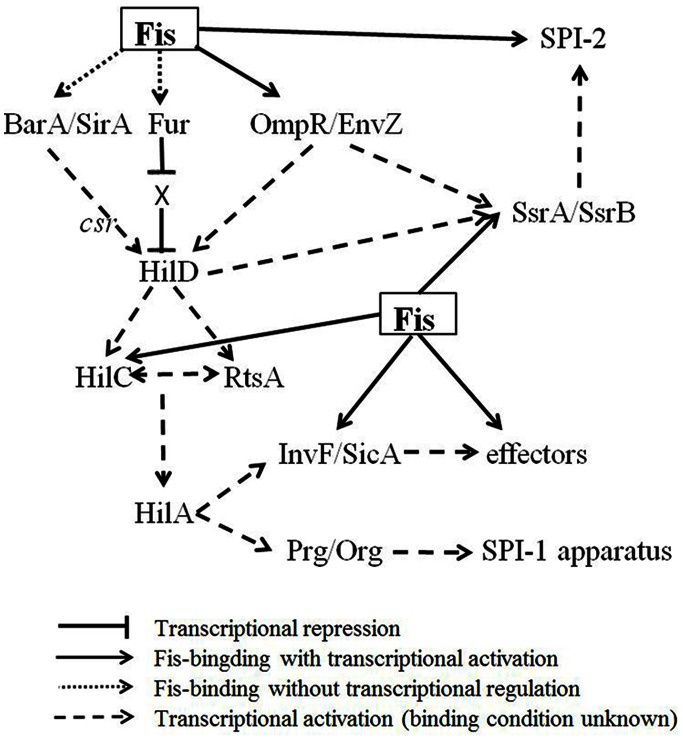
The Fis regulation network of SPI-1 and SPI-2 genes. The regulatory network between SPI genes has been reported previously [25], [32], we provide the effect of Fis in this network.

Fis was reported to influence the expression of many genes on SPI-1 and SPI-2 in *S. enterica* serovar Typhimurium. Most previous research has mainly been focused on the effects of Fis on regulating the expression of SPI genes, but the relevant molecular mechanisms are unclear. In this study, we firstly conclude the existence of three modes of Fis regulatory mechanism on SPI genes, and provide a regulatory network of Fis on SPI genes (Figure 8), including: i) Fis binds to and activates the SPI positive regulator genes; ii) Fis binds to the ORF region of SPI genes to mediate transcriptional activation of these genes and those downstream genes; iii) Fis positively influences the SPI regulators by binding to and activating *ompR*. Among these mechanisms, the most interesting discovery is that we find the effect of Fis-binding on gene ORF regions may not exert a single effect on the single corresponding gene, but a small scale effect on several genes, which was not reported before.

In most studies about global binding profiles of TFs in bacteria, such as Fis, HNS, RutR and Sfh, TF binding sites have been found to be located at both ORF regions and intergenic regions [3], [4], [13], [73]–[75]. Several studies further indicated that TF binding in ORF regions could also regulate the transcription of corresponding genes [3], [4], [73], however the exact molecular mechanism for that is still unclear [76]. We noted that a ChIP-chip analysis to determine the genomic binding profiles of σ^70^ in *E. coli* revealed the existence of many σ^70^–binding sites within the ORF regions [77], and another study suggested that at least 37 ORF regions bound by Fis in *E. coli* also have the core RNA polymerase (RNAP) or σ^70^ binding sites [4]. It has been proposed that Fis regulates the transcription by formation of DNA microloops, which form a separate topologica domain [78], and in those regions, the RNAP may be trapped to repress the transcription or may recycle to efficiently activate the gene transcription process [4]. Therefore we suggest Fis-binding on SPI genes may recycle RNAP to promote the transcription of corresponding genes and those downstream genes.

In this study, the identified Fis-dependent genes in SPI-1 and SPI-2 includes nine genes encoding effector proteins, which are transported via the T3SS into the host cell, 37 genes encoding needle complex structural proteins, six genes encoding SPI regulators, and three genes with unknown functions. Almost all genes encoding needle complex structural proteins and SPI regulators were activated by Fis. However, for the 18 genes encoding known effectors, only nine of them showed decrease expression in *Δfis*. It can be speculated that the weak effects of Fis on effectors observed in this study result from the inability of the LT2 growth conditions (Luria-Bertani broth) to induce transcription of effectors [79], [80].

Although most genes on SPI were activated by Fis, a small proportion of SPI genes were down-regulated, such as STM2911 and STM2912 in SPI-1 and *ttrB* in SPI-2. STM2912 was annotated as a putative transcriptional regulator [24], and we propose that it is likely to be a negative regulator for SPI genes, which will be the subject of future study.

## Supporting Information

Figure S1 Fis-regulated genes on SPI-1 and SPI-2.
(TIF)

Figure S2 Confirm Fis-binding sites by using gel mobility shift assays. The DNA fragments (1 nM) were incubated with increasing concentrations of Fis protein (0, 50, 100, 150, 200, 250, 300, 350, 400 nM).
(TIF)

Table S1 Strains and plasmids used in this study.
(DOC)

Table S2 Oligonucleotide primers used in this study (5′-3′).
(DOC)

Table S3 Fis binding regions detected by ChIP-seq.
(XLS)

Table S4 Validation of 20 randomly selected Fis-binding sites and 2 control sites by RT-PCR.
(DOC)

Table S5 Fis up-regulated and down-regulated genes detected by RNA-seq.
(XLS)

Table S6 Confirmation of RNA-seq results by RT-PCR.
(DOC)

Table S7 Effect of *pocR* on Fis-regulated B_12_ biosynthesis genes.
(DOC)

Table S8 The SPI genes regulated by Fis under three regulatory mechanism.
(XLS)
