# Supplementary figures and images for: Genome-Wide Analysis of the Salmonella Fis Regulon and Its Regulatory Mechanism on Pathogenicity Islands
Source: PLoS One. 2013 May 23;8(5):e64688. doi: 10.1371/journal.pone.0064688 (PMC3662779; doi:10.1371/journal.pone.0064688)

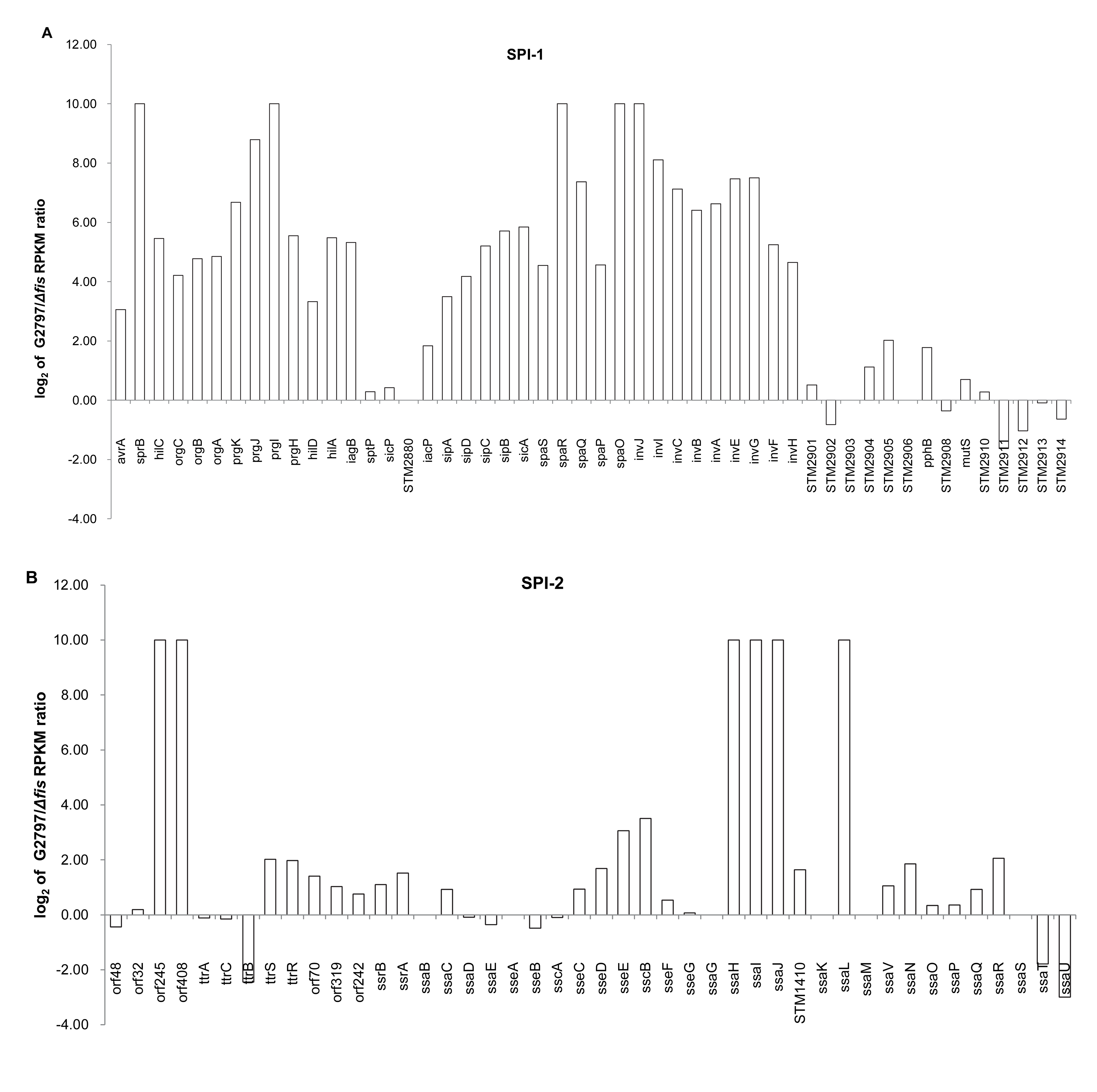

Supplement: Figure S1 — Fis-regulated genes on SPI-1 and SPI-2. (TIF) [file pone.0064688.s001.tif]

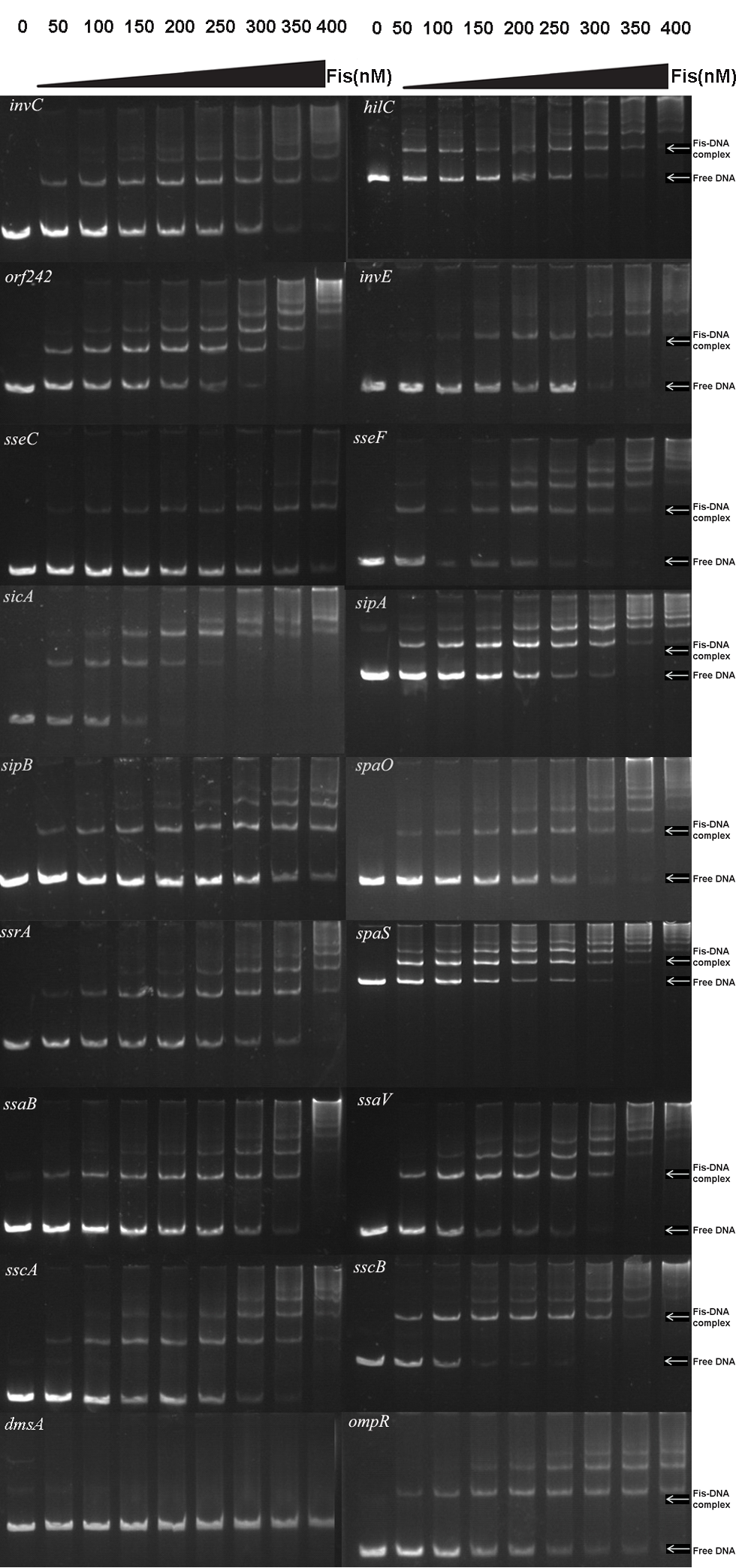

Supplement: Figure S2 — Confirm Fis-binding sites by using gel mobility shift assays. The DNA fragments (1 nM) were incubated with increasing concentrations of Fis protein (0, 50, 100, 150, 200, 250, 300, 350, 400 nM). (TIF) [file pone.0064688.s002.tif]
