# Supplementary material for: Genome-Wide Analysis of the Salmonella Fis Regulon and Its Regulatory Mechanism on Pathogenicity Islands
Source: PLoS One. 2013 May 23;8(5):e64688. doi: 10.1371/journal.pone.0064688 (PMC3662779; doi:10.1371/journal.pone.0064688)
Supplement: Table S1 — Strains and plasmids used in this study. (DOC) [file pone.0064688.s003.doc]

**Table S1: Strains and plasmids used in this study**

| **Strains** | Genotype or description | Reference |
| --- | --- | --- |
| G2797 | Wild-type *S.enterica* serovar Typhimurium LT2 | Lab collection |
| H2101 | *fis* deletion mutant in G2797,CmR | This work |
| H2102 | Encodes chromosomally FLAG-tagged *fis* in G2797, CmR | This work |
| H2103 | *ompR* deletion mutant in LT2*,*KmR | This work |
| H2104 | *ompR* and *fis* double deletion mutant in LT2*,*KanR, CmR | This work |
| H2105 | *fis* mutant, containing intergrated pWSK129-*ompR,*KmR | This work |
| H2106 | Deletion of Fis-binding site on *invE*, KmR | This work |
| H2107 | Deletion of Fis-binding site on *invC*, KmR | This work |
| H2108 | Deletion of Fis-binding site on *spaO*, KmR | This work |
| H2109 | Insert kan on the upstream of Fis-binding site on *invE*, KmR | This work |
| H2110 | Insert kan on the upstream of Fis-binding site on *invC*, KmR | This work |
| H2111 | Insert kan on the upstream of Fis-binding site on *spaO*, KmR | This work |
| G1370 | DH5а | Lab collection |
| H2112 | DH5а with pGEM-Teasy carrying 3*FLAG-cat, ApR, CmR | This work |
| H2113 | DH5а with integrated pGEX4T-1-*fis*, KmR | This work |
| G1488 | BL21 | Lab collection |
| H2114 | BL21 with integrated pGEX4T-1-*fis*, KmR | This work |
| H2188 | *flhD* deletion mutant in LT2*,*KmR | This work |
| H2189 | *fruR* deletion mutant in LT2*,*KmR | This work |
| H2190 | *fucR* deletion mutant in LT2*,*KmR | This work |
| H2191 | *gutM* deletion mutant in LT2*,*KmR | This work |
| H2192 | *pocR* deletion mutant in LT2*,*KmR | This work |
| H2193 | *prpR* deletion mutant in LT2*,*KmR | This work |
| H2194 | *flhD* and *fis* double deletion mutant in LT2*,*KanR, CmR | This work |
| H2195 | *fruR* and *fis* double deletion mutant in LT2*,*KanR, CmR | This work |
| H2196 | *fucR* and *fis* double deletion mutant in LT2*,*KanR, CmR | This work |
| H2197 | *gutM* and *fis* double deletion mutant in LT2*,*KanR, CmR | This work |
| H2198 | *pocR* and *fis* double deletion mutant in LT2*,*KanR, CmR | This work |
| H2199 | *prpR* and *fis* double deletion mutant in LT2*,*KanR, CmR | This work |
| **Plasmids** | | |
| pLW1600 | pGEM-Teasy carrying 3*FLAG-cat, ApR, CmR | This work |
| pGEX4T-1 | Expression vector, ApR | Lab collection |
| pLW1601 | pGEX4T-1, carrying LT2 *fis*, ApR | This work |
| pWSK129 | Low copy cloning vector, KmR | [1] |
| pLW1599 | pWSK129, carrying LT2 *ompR,* KmR | This work |
| pKD46 | Red recombination plasmid, ApR | Lab collection |
| pKD3 | Containing a chloramphenicol resistance cassette and the flipase recognition sites, CmR | Lab collection |
| pKD4 | Containing a kanamycin resistance cassette and the flipase recognition sites, KmR | Lab collection |

**References:**

1. Wang RF, Kushner SR (1991) Construction of versatile low-copy-number vectors for cloning, sequencing and gene expression in *Escherichia coli*. *Gene* 100: 195-199.
