# Supplementary material for: Genome-Wide Analysis of the Salmonella Fis Regulon and Its Regulatory Mechanism on Pathogenicity Islands
Source: PLoS One. 2013 May 23;8(5):e64688. doi: 10.1371/journal.pone.0064688 (PMC3662779; doi:10.1371/journal.pone.0064688)
Supplement: Table S2 — Oligonucleotide primers used in this study (5′-3′). (DOC) [file pone.0064688.s004.doc]

**Table S2: Oligonucleotide primers used in this study (5'-3')**

| MUTANT CONSTRUCTION |  | |
| --- | --- | --- |
| *Deletion mutants* | | |
| *Δfis::Cm*-pKD3 | F | ATGTTCGAACAACGCGTAAATTCTGACGTACTGACCGTAGCTTCGACGAGATTTTCAGG |
| *Δfis::Cm*-pKD3 | R | TTAGTTCATGCCGTATTTTTTTAATTTTTTACGCAGCGCACTTATTCAGGCGTAGCAC |
| *ΔspaO::Km*-pKD4 | F | GAAAAAGTTAGGTCATTTCAACCGTGTTGAAGGGGGAATGATTGAACAAGATGGATTGC |
| *ΔspaO::Km*-pKD4 | R | GCTGCCCCATGGCTTCGAGTTCGGCGAGGGTAACGTTTCAGAAGAACTCGTCAAGAAGG |
| *ΔinvC::Km*-pKD4 | F | TGCGTTATCGGCGTGGGTGGGATACTCGGTATTAGGCATGATTGAACAAGATGGATTGC |
| *ΔinvC::Km*-pKD4 | R | CACAGGTCAATAACCCGTCAATCGCGCGCACACCGGTTCAGAAGAACTCGTCAAGAAGG |
| *ΔinvE::Km*-pKD4 | F | ACTACTGATGCTTTCGCTATTGCAACAGCCACATGAAATGATTGAACAAGATGGATTGC |
| *ΔinvE::Km*-pKD4 | R | GCCAATATTCTTCATAAAAGAGTGAAGAGGGTATGGCTCAGAAGAACTCGTCAAGAAGG |
| *ΔompR::Km*-pKD4 | F | CACTTACATTTGTTGCGAACCTTTGGGAGTACAGACAATGATTGAACAAGATGGATTGC |
| *ΔompR::Km*-pKD4 | R | CGGGCAAATGAACTTCGCGGTGAGAAGCGCATTCGCCTCAGAAGAACTCGTCAAGAAGG |
| *ΔflhD::Km*-pKD4 | F | CGGCTACGTCGCACAAAAATAAAGTTGGTTATTCTGGGTGTAGGCTGGAGCTGCTTC |
| *ΔflhD::Km*-pKD4 | R | ATCGCGAGCTTCCTGAACAATGCTTTTTTCACTCATTAATGGGAATTAGCCATGGTCC |
| *ΔfruR::Km*-pKD4 | F | AAAGGTTATGGTTTGTACAATTTACACAAGGGGCAATATGGGAATTAGCCATGGTCC |
| *ΔfruR::Km*-pKD4 | R | GACGGCGGCAGAAGAGAGAGTCTTTTACCGCCGGTCCTGTGTAGGCTGGAGCTGCTTC |
| *ΔgutM::Km*-pKD4 | F | CGGTTACGCGCCATCAGGCAAAACAACAGGAGCACATTGTGTAGGCTGGAGCTGCTTC |
| *ΔgutM::Km*-pKD4 | R | AATGATAATTTCGCAAGCTATACGCATTCACAACGAAAATGGGAATTAGCCATGGTCC |
| *ΔpocR::Km*-pKD4 | F | TTTTGTTTATAACAATAAATTAACTGAGGGGTTTTATCGTGTAGGCTGGAGCTGCTTC |
| *ΔpocR::Km*-pKD4 | R | AAGACTATCAAAAATCGGCAATAGCAAAATATTGCTATATGGGAATTAGCCATGGTCC |
| *ΔprpR::Km*-pKD4 | F | AAAACAAAAAATCGCCCCTTAAAGATGAATCTACTGCGTGTAGGCTGGAGCTGCTTC |
| *ΔprpR::Km*-pKD4 | R | CGTAGCGCCATCAGGCATTCAGGACGCTGTCATTTGTCATGGGAATTAGCCATGGTCC |
| *ΔfucR::Km*-pKD4 | F | CATGACGCGGGCGTTCATCGAATGATGGGGTGAGAAAAGTGTAGGCTGGAGCTGCTTC |
| *ΔfucR::Km*-pKD4 | R | GCGCGTCATCAGCGCCGGATGACGCACGCTTATCCGGTATGGGAATTAGCCATGGTCC |
| *Insertion mutants* | | |
| *fis*-FLAG-PGEM-T-3F | F | GTACGCTGCGTAAAAAATTAAAAAAATACGGCATGAAC GACTACAAAGACCATGACGG |
| *fis*-FLAG-PGEM-T-3F | R | AGTAGCGCCTTTTTAAACAAGCAGTTAGCTAATCGAAA CACTTATTCAGGCGTAGCAC |
| *spaO*-Km-pKD4 | F | GAAAAAGTTAGGTCATTTCAACCGTGTTGAAGGGGGAATGATTGAACAAGATGGATTGC |
| *spaO*-Km-pKD4 | R | CTTCGATATGTTGAATATCTAACGTTTCCACAATAATTCAGAAGAACTCGTCAAGAAGG |
| *invC*-Km-pKD4 | F | TGCGTTATCGGCGTGGGTGGGATACTCGGTATTAGGCATGATTGAACAAGATGGATTGC |
| *invC*-Km-pKD4 | R | AACGCTCAACGATTTTCCCTGTCGGATCCAACACCGCTCAGAAGAACTCGTCAAGAAGG |
| *invE*-Km-pKD4 | F | ACTACTGATGCTTTCGCTATTGCAACAGCCACATGAAATGATTGAACAAGATGGATTGC |
| *invE*-Km-pKD4 | R | CATTCAAACCTATAATATCGGCTAACAGCGAATCCACTCAGAAGAACTCGTCAAGAAGG |
| CLONING | | |
| pwsk-129-*ompR* | F | CGGGATCCATGCAAGAGAATTATAAGATTC |
| pwsk-129-*ompR* | R | GGAATTCTCATGCTTTAGAACCGTCCGGT |
| QUANTITATIVE PCR | | |
| *Gene expression* | | |
| *sipC* | F | AATAATGTCACGACTAAAGCGAATG |
| *sipC* | R | CGCAACGGCACTGGAAGA |
| *gntT* | F | GCGCCATGCTGGGCAAAATGCT |
| *gntT* | R | CGCTGCGGCAATGGTGAAT |
| *hilC* | F | CCAAAGTATTTTTTTCATGCGG |
| *hilC* | R | GCTCAAGGAAATCAAACCCAC |
| *rpoS* | F | AGATTGGGTATTCACCACTGTTAA |
| *rpoS* | R | GTCCAGCAACGCTTTTTCGGA |
| *entB* | F | CTTTTGGGGCCGCAACTGCCCAA |
| *entB* | R | CCGCCACCATAAACGGCTT |
| *invF* | F | TGTCGCACCAGTATCAGG |
| *invF* | R | ATAGTCTTCTCCCAGCAT |
| *pgtE* | F | CGCCACGTTGCAGGGGGA |
| *pgtE* | R | CCAATATATCGACCATTAT |
| *phoP* | F | TGTAGAGGATAATGCAT |
| *phoP* | R | CGCATTAACGCCTGCATACG |
| *hilA* | F | TCTTACCCGCTGTATTTATGCC |
| *hilA* | R | TCTGAAAAGGAAGTATCGCCAAT |
| *wecD* | F | TAAAAGTGCCGGCTGAAAACA |
| *wecD* | R | CACCAGGCATTGGTGATCA |
| *sseJ* | F | ACACCGGTACAGGTTCGCGGA |
| *sseJ* | R | CCTTCCGCCAAAGTATTG |
| *ssaR* | F | ACTGATTGGTATATTGT |
| *ssaR* | R | ATTCCGAAAATAATTGGC |
| *invA* | F | GACCACGGTGACAATAGAGAAGAC |
| *invA* | R | CGCTCTTTCGTCTGGCATTA |
| *invB* | F | TGTTCTGGATTTATTTGCATTGC |
| *invB* | R | TTCATAAGCCCGCTGTTGTAAT |
| *invC* | F | GCGACAACCGTAGCGGAATA |
| *invC* | R | TCCAGCAGTACCGTATAAAAGGC |
| *invI* | F | ATCCTTGAACAAATAGCGGGTC |
| *invI* | R | CGGCGAACAATAGACTGCTTAC |
| *invJ* | F | GAAGTAACCGATAACGCCACG |
| *invJ* | R | CGATAGTGGTTGGTTGCAGC |
| *spaO* | F | CAGAGCGACCGTTTGAGTTG |
| *spaO* | R | TGCAGGCAGTTCAGGAAGATG |
| *spaP* | F | TTTCCACCCTGTTGCCATTT |
| *spaP* | R | CACATAACAAACATAGAAAGCAGCA |
| *spaQ* | F | TGGTAGGGTTATTCCAGACGGT |
| *spaQ* | R | TGACGCCCGTAAGAGAGTAAAAC |
| *spaR* | F | CCTGGTGGCATTGGGAGTAT |
| *spaR* | R | GATACTACTACTTAGCGTTGCCCC |
| *spaS* | F | TGTAGGTATTGCCGTCATTTGG |
| *spaS* | R | ACGCTTCACTTCTTCCTTATCCA |
| *hilD* | F | TTTCACTTTAGTTTGCTTTCGGA |
| *hilD* | R | GATAAGTGCATAGAGAGCGCCA |
| *prgH* | F | TGGAAACATCAAAAGAGAAGACG |
| *prgH* | R | ATCGGCAGGTATATCAGGGAGT |
| *ssrB* | F | CGAGCCTGACATACTTATCCTTG |
| *ssrB* | R | CCGCTAACAGAACTTGCTGACTAC |
| *ssrA* | F | CGGCTGGTATTCTTGTAAGGGT |
| *ssrA* | R | AAGCAGACACAAATTCGCAAG |
| *fruK* | F | ATGGTCTGCGTCAGCGGTAG |
| *fruK* | R | TCGGTAATACCCACATTGCTCT |
| *eno* | F | TGACGAAGGCGGCTATGC |
| *eno* | R | AGAACGTATTTACCGTCTTTGTAG |
| *cydA* | F | GCTGAATGGGAAACGCAACC |
| *cydA* | R | CCAGCAGTTCATAGGCTTTCATC |
| *marR* | F | AAATCATTCCGCTGGGTCG |
| *marR* | R | CATTAGGATTCGGCAGTCTTT |
| *fucI* | F | AGGCATTCCAGCGTTCTCC |
| *fucI* | R | GCCCAGCCAGGATTCAAAGA |
| *fucK* | F | TAACACGCTGTATAAACTGGTCTGG |
| *fucK* | R | GCGTCTGTCTGTAGCGTGCC |
| *fucO* | F | TATCGCCGACCCGTAAACC |
| *fucO* | R | CGGCCCGGGTAATATAGC |
| *flgD* | F | AGAACCAGGACCCGACTAACC |
| *flgD* | R | TCCGTTTGCTTACCATCCC |
| *flgE* | F | ATCACCATTCCGAACACGC |
| *flgE* | R | CGCATCACTCACGCTAAAGG |
| *flgF* | F | CAGGACGGCTGGCTGGTAGT |
| *flgF* | R | TGCCTTCCGCTTTGACCAG |
| *flgG* | F | GGAACCTGTCGCAGACCAAC |
| *flgG* | R | CCCTGCTGGGTAACGCTGA |
| *flgC* | F | TGCCGCACAGTCCAAACG |
| *flgC* | R | CGGATTGCCTGGCTCATAAA |
| *srlA* | F | CGGAGTGGTTCATCGGGTTA |
| *srlA* | R | GCAGGTAACGAGATAGCGGATTT |
| *srlD* | F | CCGCTCGCTACAGGTGAAT |
| *srlD* | R | GCAGCAGTGACTGGAACATCG |
| *srlR* | F | AACCGTCATCCGCACCTA |
| *srlR* | R | AAAGCAACGGCACCATCT |
| *prpB* | F | GCGCTATCAATGCCAACCAT |
| *prpB* | R | AGCGGCAGCGGGCAAACAT |
| *prpC* | F | TTTACTGGTATCACTACAGCCACAAC |
| *prpC* | R | GCTCGGCATACAGCACCAG |
| *prpD* | F | ACTGCGGTGGTCGCTGAA |
| *prpD* | R | GTAGGAGCCGTAAGGACGC |
| *prpE* | F | CCAGTTGGGTAACGGAGACG |
| *prpE* | R | AGCGACCAGTAAGTCTTCACAAAA |
| *cbiH* | F | CGGAAATCGTCGTCGGTTAT |
| *cbiH* | R | CGGAATAAGGCGAACCTCTACAT |
| *cbiQ* | F | CACGGGTCGCCGAAACTATC |
| *cbiQ* | R | CCACCGCCTCATCCAGAAGAA |
| *cbiO* | F | TCGTTATCAGAATGAGCCGG |
| *cbiO* | R | ATAAATAAGATCGATATCAT |
| *cbiN* | F | GTTGATGTTGTTAGCGATGGTG |
| *cbiN* | R | ATCTGGCTTTCCGCTTCG |
| *cbiM* | F | AGTTGTTCACATCCGACCGG |
| *cbiM* | R | AGGCCATTTTCCACACCAGATA |
| *cbiL* | F | GTTGCCATTTCCCTATGAGCG |
| *cbiL* | R | GCATCGCCCAGGGTAATAAAA |
| *cbiK* | F | GCTTGCCGCACAGGGATA |
| *cbiK* | R | AGTGAATAACGGACGCAAGAGTT |
| *cbiJ* | F | GGTCAGCCATAACCTCCTTCG |
| *cbiJ* | R | GCGGATGCGTCAGATTGCT |
| *cbiG* | F | ATGTCATTAGCCTGCTTTCCG |
| *cbiG* | R | ACATCTCATTGACATCCGTTGC |
| *cbiF* | F | GCAGGCAGAACGCTACGAC |
| *cbiF* | R | TAACTCCTCGCCTTGCTCAC |
| *cbiT* | F | ATCGGCGCATGTCGAATG |
| *cbiT* | R | TTTCTTCCTTCTGACAGGCGATA |
| *cbiE* | F | TTGGCGGCGAACGATTTA |
| *cbiE* | R | GCGTGTACCGATGCCATAAAA |
| *cbiD* | F | CATTTCCATTATTGGCACTACCG |
| *cbiD* | R | TTTCCAGGATGACCCACCAG |
| *cbiC* | F | ACAGTTTCACCATTATTAGCGACAT |
| *cbiC* | R | GAAATCGGCGGTGGTATGAA |
| *cbiB* | F | ACCTTGCGGAAAGCCGAATA |
| *cbiB* | R | GCCGCCGAGAAAGAGGAAA |
| *cbiA* | F | TGCTTCCTCCTCCTGTTCTCA |
| *cbiA* | R | CTTGCCGTCCACCAGTAGG |
| *Chromatin immunoprecipitation* | | |
| *hupA.ChIP* | F | AAAGAAGGCGATGCTGTACAAC |
| *hupA.ChIP* | R | TTTAGTTTTAGCGGCGGC |
| *sipC.ChIP* | F | CAGTTTGCTATCAGCCTGGTTC |
| *sipC.ChIP* | R | GTGCAAACCCAGTTACGCG |
| *hin.ChIP* | F | GTATTGATACCAGTAGCGCGATG |
| *hin.ChIP* | R | ATGTTTGTTGATCGCCCGAG |
| *carA.ChIP* | F | ATCCGGCTTAACTTCAGAATGC |
| *carA.ChIP* | R | CCGTCTTCCAGAACCAATAGC |
| *fis.ChIP* | F | GCAGTACCCGGATTTAGTGAAGT |
| *fis.ChIP* | R | CAGTTGGGCAATCTCTACGC |
| *nrfA.ChIP* | F | TGGATGAGACCTCTATGGCAAG |
| *nrfA.ChIP* | R | TTGCTCAGCGTAAACAGAGGTAA |
| *rrsA.ChIP* | F | TGACTCTGAAGCGGGAAAGC |
| *rrsA.ChIP* | R | CGTATCTTCGAGTGCCCACA |
| *tgt.ChIP* | F | AGCCTTTATGCCTGTGGGTACT |
| *tgt.ChIP* | R | GTGGAAAGTGTTGCCGAGAATA |
| *dps.ChIP* | F | TCGGCCAGTTCTTTTAAGTGATC |
| *dps.ChIP* | R | CACGCAAGTTATCAACAGCAAA |
| *ompR.ChIP* | F | ACCCCATTATTTATTCTGTCGG |
| *ompR.ChIP* | R | TGAGCTCTTGTTTGAGTGTTTCG |
| *sipB.ChIP* | F | TTTCGTTGCCACCACATCTTTA |
| *sipB.ChIP* | R | TGACGCAAGTAGCATTAGCCG |
| *sscB.ChIP* | F | GGATGCCAGCCATCCAGA |
| *sscB.ChIP* | R | ATTGCGGTTTGAAAAGCCTC |
| *ssaU.ChIP* | F | GCATTCTACCGAAACACCATAAA |
| *ssaU.ChIP* | R | GTGCTTGTGCCAGGCTAAAA |
| *mgtC.ChIP* | F | AATGCTCCAGTGAATTGCGG |
| *mgtC.ChIP* | R | CGCTCAGGAACAGGGCTATAA |
| *sopB.ChIP* | F | TGTTGGTATAGTGATGCCCGTT |
| *sopB.ChIP* | R | CTTTGCGGCAGTAGCGTTTAG |
| *STM2332.ChIP* | F | CGGCGATAAAAAACCGTAAATT |
| *STM2332.ChIP* | R | TCCTGGGCTATCTGGGAATTAA |
| *STM0212.ChIP* | F | TGATGGTCGTGGAATATGCTG |
| *STM0212.ChIP* | R | CCGTGAGTCCTCCTGTTTGC |
| *ptsG.ChIP* | F | GGCTTTGGTTGCGGCGTTTG |
| *ptsG.ChIP* | R | TAGCCTGAACGCCGGAACCC |
| *STM1250.ChIP* | F | GTCTTACCGCCAGTTGCT |
| *STM1250.ChIP* | R | CAACGCCCTTCTCCATTA |
| *invC.ChIP* | F | ATCAGCGGTTCACGGACGCCAACG |
| *invC.ChIP* | R | TGCGTTATCGGCGTGGGTGG |
| *metJ.ChIP* | F | GCGTTGTGCCGCCAATCCA |
| *metJ.ChIP* | R | CGCCTTCCAGTTCCGCTAAT |
