# Supplementary material for: Genome-Wide Analysis of the Salmonella Fis Regulon and Its Regulatory Mechanism on Pathogenicity Islands
Source: PLoS One. 2013 May 23;8(5):e64688. doi: 10.1371/journal.pone.0064688 (PMC3662779; doi:10.1371/journal.pone.0064688)
Supplement: Table S4 — Validation of 20 randomly selected Fis-binding sites and 2 control sites by RT-PCR. (DOC) [file pone.0064688.s006.doc]

**Table S4:** Validation of 20 randomly selected Fis-binding sites and 2 control sites by RT-PCR.

| **Gene number** | **Gene name** | **Type^a^** | **B-site** | **Peak Start^b^** | **Peak End^c^** | **ChIP-seq^d^** | **RT-PCR^e^** | **Reported^f^** |
| --- | --- | --- | --- | --- | --- | --- | --- | --- |
| STM0212 |  | ORF | 41 | 248344 | 248785 | ∞^h^ | 3.44 |  |
| STM0405 | *tgt* | ORF | 74 | 460511 | 460995 | 4.69 | 1.66 | √ |
| STM0831 | *dps* | IG1 | 132 | 897864 | 898448 | 3.42 | 1.04 | √ |
| STM1044 | *sodC* | IG1 | 170 | 1130437 | 1130877 | 6.06 | 0.58 | √ |
| STM1091 | *sopB* | IG3 | 181 | 1179137 | 1179922 | 1.43 | 5.28 |  |
| STM1203 | *ptsG* | IG2 | 205 | 1287599 | 1288336 | 6.40 | 5.46 |  |
| STM1250 |  | IG3 | 215 | 1335116 | 1335620 | 6.89 | 22.76 |  |
| STM1403 | *sscB* | ORF | 251 | 1487757 | 1487856 | ∞ | 3.40 |  |
| STM1422 | *ssaU* | IG3 | 257 | 1501436 | 1501649 | 6.00 | 2.84 |  |
| STM2332 |  | IG3 | 402 | 2443160 | 2443349 | ∞ | 2.14 |  |
| STM2772 | *hin* | ORF | 486 | 2915017 | 2915414 | 7.07 | 0.47 | √ |
| STM2884 | *sipC* | ORF | 506 | 3028658 | 3029086 | 4.89 | 2.75 |  |
| STM2885 | *sipB* | IG3 | 507 | 3030713 | 3030920 | ∞ | 1.68 |  |
| STM2894 | *invC* | ORF | 511 | 3037560 | 3037694 | ∞ | 13.62 |  |
| STM3380 | *accC* | ORF | 617 | 3551463 | 3551903 | 3.69 | 5.49 |  |
| STM3502 | *ompR* | IG3 | 643 | 3660247 | 3660669 | 4.89 | 1.54 |  |
| STM3764 | *mgtC* | ORF | 703 | 3964495 | 3964639 | 0.26 | 4.82 |  |
| STM3988 | *rrsA* | IG1 | 741 | 4195823 | 4196003 | ∞ | 1.44 | √ |
| STM4099 | *metJ* | IG1 | 769 | 4309335 | 4309655 | 5.19 | 0.89 |  |
| STM4277 | *nrfA* | IG3 | 809 | 4516068 | 4516167 | 2.81 | 2.30 | √ |
| STM4170 | *hupA* | NA^g^ | NA | NA | NA | NA | -0.23 |  |
| STM0066 | *carA* | NA | NA | NA | NA | NA | -2.70 |  |

^a^ The coordinate position of Fis-binding site to genes.

^bc^ Obtained from NC_003197 build of the *S*. *enterica* serovar Typhimurium LT2.

^d^ Log_2_ of enrichment ratio (ratio between IP and mock-IP samples) obtained from Fis ChIP-seq .

^e^ Log_2_ of enrichment ratio (ratio between IP and mock-IP samples) obtained from the quantitative PCR.

^f^ Whether the Fis-binding site was reported previously or not.

^g^ The value is not available or undetectable.

^h^ The RPKM value of the region in mock-IP sample is undetectable.
