# Supplementary material for: Genome-Wide Analysis of the Salmonella Fis Regulon and Its Regulatory Mechanism on Pathogenicity Islands
Source: PLoS One. 2013 May 23;8(5):e64688. doi: 10.1371/journal.pone.0064688 (PMC3662779; doi:10.1371/journal.pone.0064688)
Supplement: Table S6 — Confirmation of RNA-seq results by RT-PCR. (DOC) [file pone.0064688.s008.doc]

**Table S6: Confirmation of RNA-seq results by RT-PCR**.

| **Gene number** | **Gene name** | **Orientation^a^** | **RNA-seq^b^** | **RT-PCR^c^** |
| --- | --- | --- | --- | --- |
| STM2884 | *sipC* | - | 5.21 | 5.56 |
| STM3512 | *gntT* | + | 0.01 | 0.35 |
| STM2867 | *hilC* | - | 5.45 | 6.39 |
| STM2924 | *rpoS* | - | -0.80 | -1.29 |
| STM0597 | *entB* | + | -3.69 | -4.25 |
| STM2899 | *invF* | - | 5.25 | 1.01 |
| STM2395 | *pgtE* | - | 0.55 | 1.29 |
| STM1231 | *phoP* | - | 1.21 | 1.42 |
| STM2876 | *hilA* | + | 5.48 | 5.33 |
| STM3924 | *wecD* | + | -0.65 | -0.59 |
| STM1631 | *sseJ* | + | NA^d^ | 0.02 |
| STM1419 | *ssaR* | + | 2.05 | 0.63 |

^a^ The transcription direction (+: Watson strand, -: Crick strand).

^b^ Log_2_ of expression ratios (ratio of RPKM in wild-type and *fis* deletion mutant samples) obtained from RNA-seq.

^c^ Log_2_ of expression ratios (ratio of RPKM in wild-type and *fis* deletion mutant samples) obtained from the RT-PCR.

^d^ The RPKM in wild-type is undetectable (approximately 0).
