# Supplementary material for: Genome-Wide Analysis of the Salmonella Fis Regulon and Its Regulatory Mechanism on Pathogenicity Islands
Source: PLoS One. 2013 May 23;8(5):e64688. doi: 10.1371/journal.pone.0064688 (PMC3662779; doi:10.1371/journal.pone.0064688)
Supplement: Table S7 — Effect of pocR on Fis-regulated B12 biosynthesis genes. (DOC) [file pone.0064688.s009.doc]

**Table S7. Effect of *pocR* on Fis-regulated B_12_ biosynthesis genes**

| **B_12_ biosynthesis genes** | **log_2_(wt/*Δfis*)^a^** | **log_2_(*ΔpocR*/*ΔpocRΔfis*)^b^** |
| --- | --- | --- |
| *cbiN* | -0.65 | 0.42 |
| *cbiM* | 0.50 | 0.55 |
| *cbiL* | 0.16 | 0.85 |
| *cbiK* | -0.04 | 0.50 |
| *cbiJ* | -0.72 | 0.55 |
| *cbiG* | -0.03 | 1.28 |
| *cbiF* | 0.72 | 0.79 |
| *cbiT* | -0.73 | 1.17 |
| *cbiE* | -1.19 | 0.68 |
| *cbiD* | 0.15 | 1.08 |
| *cbiC* | -1.77 | 0.77 |
| *cbiB* | -2.15 | 1.00 |
| *cbiA* | -0.74 | 0.69 |

^a^ Log_2_ of enrichment ratio (ratio between LT2 wild-type and *Δfis*) obtained from RT-PCR*.*

^b^ Log_2_ of enrichment ratio (ratio between *ΔpocR* and *ΔpocR/ΔpocRΔfis*) obtained from RT-PCR*.*
